# Supplementary material for: A Novel Clinical Nomogram for Predicting Overall Survival in Patients with Emergency Surgery for Colorectal Cancer
Source: J Pers Med. 2023 Mar 24;13(4):575. doi: 10.3390/jpm13040575 (PMC10145637; doi:10.3390/jpm13040575)
Supplement: Supplementary file 1 [file jpm-13-00575-s001.zip › jpm-2261710-supplementary.pdf]

Supplementary material

Explanatory data included ROC curve figures for age, NLR, PLR, LMR, PNI, Charlson score, age-adjusted Charlson score, calibration plots for 1-year overall survival, 3-years overall survival, 5-years overall survival years and nomogram for predicting overall survival using pTNM.

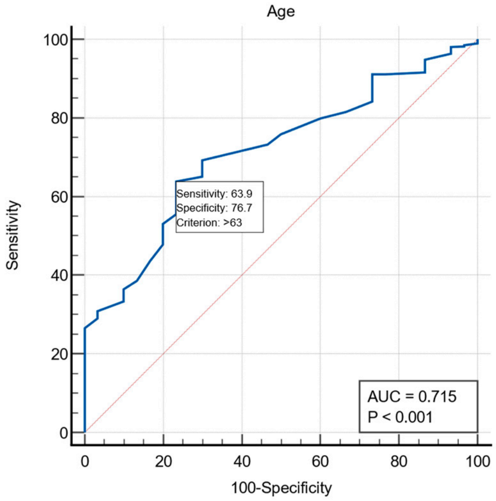

Supplementary Figure S1. ROC curve and cut-off value for age.

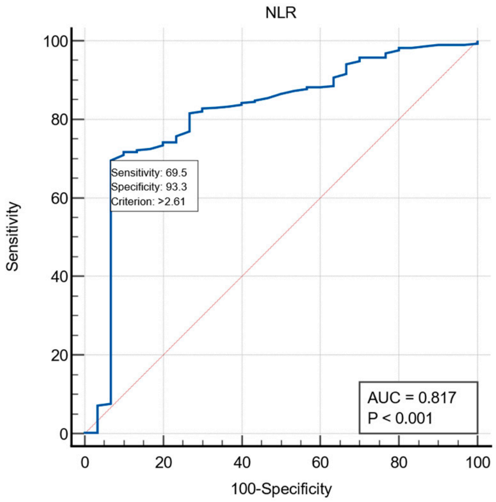

Supplementary Figure S2. ROC curve and cut-off value for NLR.

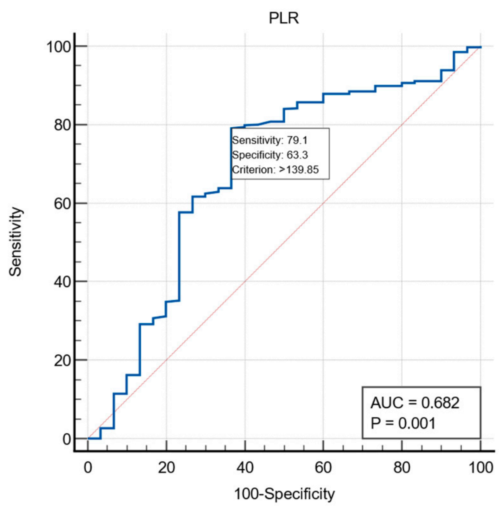

Supplementary Figure S3. ROC curve and cut-off value for PLR.

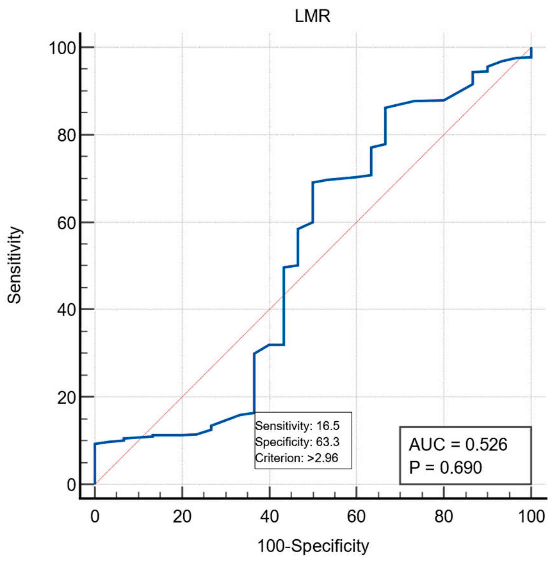

Supplementary Figure S4. ROC curve and cut-off value for LMR.

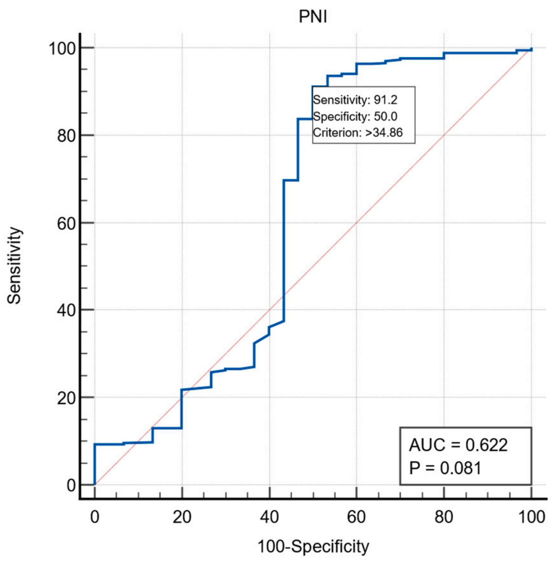

Supplementary Figure S5. ROC curve and cut-off value for PNI.

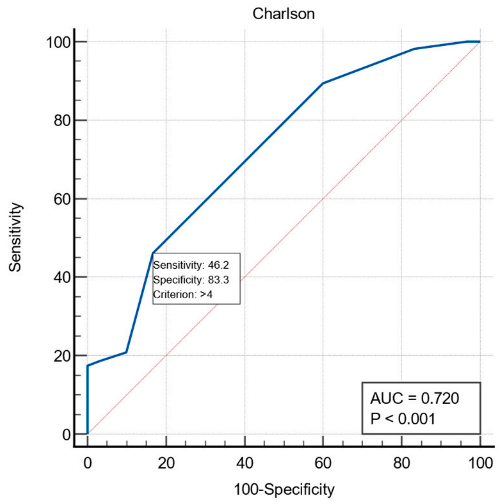

Supplementary Figure S6. ROC curve and cut-off value for Charlson score.

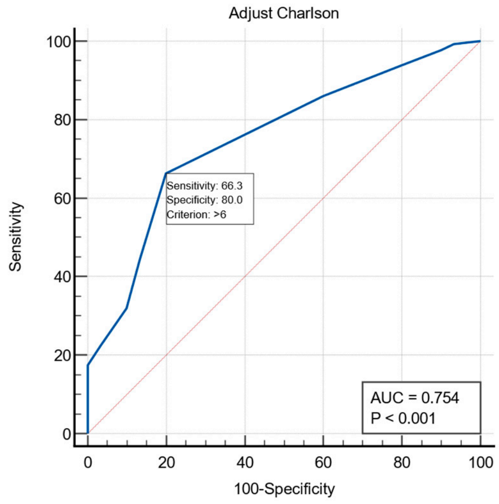

Supplementary Figure S7. ROC curve and cut-off value for age-adjusted Charlson score.

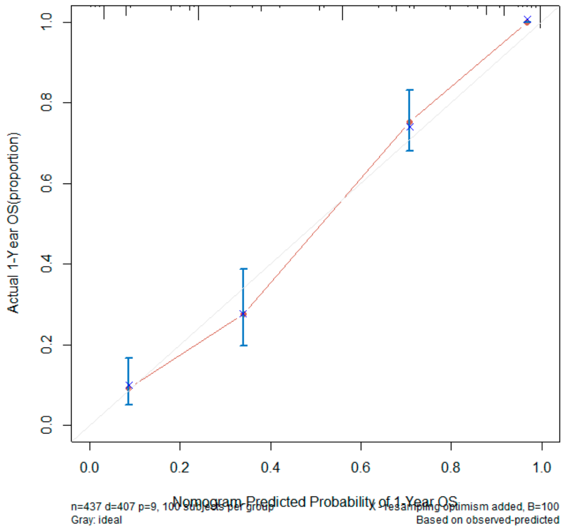

Supplementary Figure S8. 1-year overall survival nomogram calibration plot.

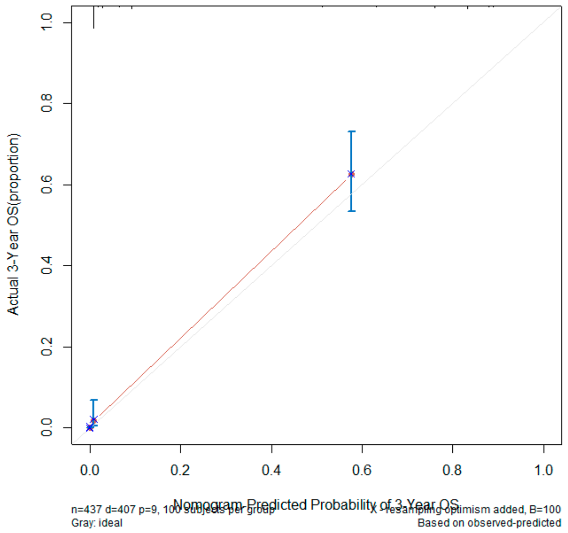

Supplementary Figure S9. 3-years overall survival nomogram calibration plot.

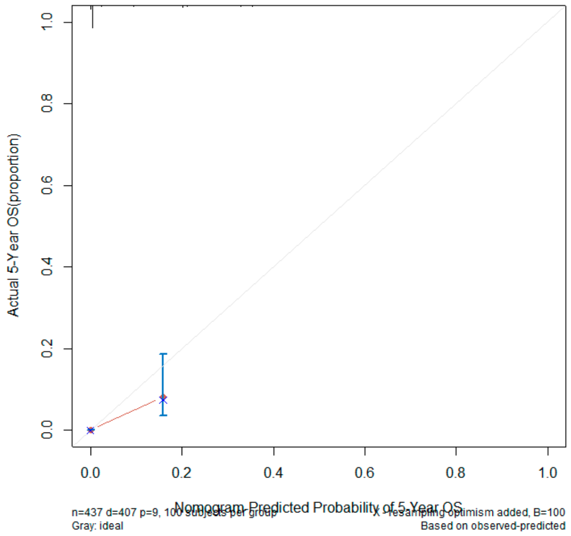

Supplementary Figure S10. 5-years overall survival nomogram calibration plot.

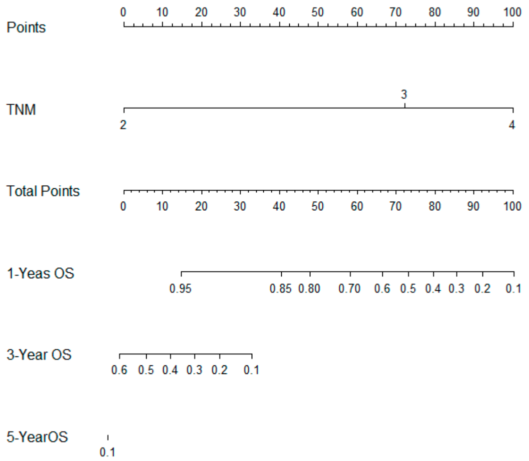

Supplementary Figure S11. Nomogram for predicting overall survival using pTNM
